# Qualitative Behavioural Assessment as a Method to Identify Potential Stressors during Commercial Sheep Transport

**DOI:** 10.3390/ani8110209

**Published:** 2018-11-15

**Authors:** Teresa Collins, Catherine A. Stockman, Anne L. Barnes, David W. Miller, Sarah L. Wickham, Patricia A. Fleming

**Affiliations:** School of Veterinary & Life Sciences, Murdoch University, Murdoch WA 6150, Australia; c.stockman@murdoch.edu.au (C.A.S.); a.barnes@murdoch.edu.au (A.L.B.); D.Miller@murdoch.edu.au (D.W.M.); sarahlwickham@outlook.com (S.L.W.); t.fleming@murdoch.edu.au (P.A.F.)

**Keywords:** qualitative behavioural assessment, QBA, sheep, transport, behaviour

## Abstract

**Simple Summary:**

Land transport is a common and unavoidable experience for most livestock, yet it remains a health and welfare concern. From the animals’ perspective, transport involves mixing with other animals, novel experiences, and prolonged standing, often after periods of water and feed withdrawal (‘curfews’). Although the physical effects of transport have been studied, often by the impact on meat quality, the effects on the mental well-being of sheep are unknown. The aim of this study was to identify factors that influence the behavioural expression of sheep undergoing land transport, using observers who were blinded to the experimental treatments to score the animal’s body language during land transport. Various vehicle crate types, deck positions, sheep breeds and point of origin were compared. All treatments were variations on current commercial transport, and therefore stocking density was similar between the vehicles as per regulatory requirements, but truck designs varied. This study supports using the scoring of behavioural expression to assess sheep welfare during transport.

**Abstract:**

Land transport is an unavoidable experience for most livestock, yet there is limited research comparing animal welfare under different conditions. We video recorded sheep responses during short (2 h) commercial road transport journeys. Using Qualitative Behavioural Assessment, observers (blinded to the treatments) scored the behavioural expression of sheep and reached significant consensus in their scoring patterns (*p* < 0.001). There were also significant effects of vehicle crate design (sheep transported in a ‘standard’ crate were more *calm*/*relaxed* than those transported in a ‘convertible’ crate), deck position (sheep on upper decks were more *curious*/*alert* than those on lower decks), and sheep breed (fat-tail sheep were more *agitated*/*distressed* than merino sheep) on observer scores. We only found marginal differences for sheep originating from feedlot or saleyard. Significant effects of vehicle driver (included as a random factor in all but one of our analyses) suggest driving patterns contributed to demeanour of the sheep. Finally, the fourteen drivers who participated in the study were asked their opinions on livestock transport; none of the factors we tested were identified by drivers as important for sheep welfare during transport. This study supports the use of qualitative measures in transport and revealed differences that could inform truck design.

## 1. Introduction

There is strong public interest and much research aimed at ensuring the welfare of transported livestock is optimal. The wellbeing of sheep during road transport can be influenced by several factors, including loading and unloading [1], stocking density [2,3], temperature and humidity [4,5], driving behaviour [6], vibration and noise, change in acceleration and cornering [4,6] road type [7] prolonged standing, unfamiliar mixing, novel environment, hunger, thirst and fatigue [4]. Additional factors, such as design of the vehicle and/or breed or past experience of the animals, may be less obvious in terms of their impacts on animal wellbeing but are nevertheless important to consider when striving for industry best practice.

Much research has examined the physiological responses of sheep to varying transport conditions. For example, increased cortisol and heart rate have been recorded for longer transport trips with extended curfew periods [8] and frequent changes in acceleration [9]. This research indicates the stress associated with some major transport risk factors, but is invasive, hence there is still a demand to develop practical assessment measures that can be applied in the field. Additionally, there are few measurable parameters that reflect the affective state, or how an animal is coping over time that do not add further distress to the individual upon collection.

Qualitative Behavioural Assessment (QBA) is an on-farm assessment tool [10,11,12] and has been shown to be a meaningful indicator for on-farm welfare in sheep [13]. QBA examines the expressive body language of animals as they interact with their environment; animals are scored against qualitative terms (e.g., *anxious*, *fearful*, *calm*) that describe these interactions. Qualitative behaviour explores not *what* the animal is doing, but *how* the animal is performing a specific behaviour, and it can be argued that stockpersons routinely use such expressive terms to manage their animals’ welfare state. QBA can therefore provide animal welfare-relevant measures that are non-invasive, relatively quick, and inexpensive, that can be applied in production scenarios where intensive animal measures are difficult to implement. The method has been validated under a range of experimental conditions (reviewed by [14]) and QBA scores correlate well with physiological stress responses of both sheep [15,16] and cattle [17,18] under experimental conditions during road transport. Furthermore, observers, blinded to treatments are able to distinguish between treatment groups based on the animals’ behavioural expression [14], demonstrating that QBA is a repeatable, objective and valid measure of animal behavioural responses.

Two important factors that influences welfare of transported animals are the characteristics of the vehicle itself and the way the vehicle is driven [19]. Vehicle design varies in response to environmental conditions and may impact ventilation system, suspension and flooring. In Australia, livestock are frequently transported for long distances by multi-decked open-sided trucks, relying on free ventilation due to vehicle movement. Poor ventilation may lead to heat stress, particularly in hot climates, as temperature inside the vehicle may rise within the standing vehicles (i.e., during loading) [20]. Poor suspension can affect animal welfare, as excessive vibration has been shown to lead to fear and muscle fatigue in calves [21]. Non-slippery flooring is essential to prevent animals falling, and vehicle movement and driving quality may cause distress to sheep as animals are forced to continually balance the effect of movement forces [22].

**Figure 1:**
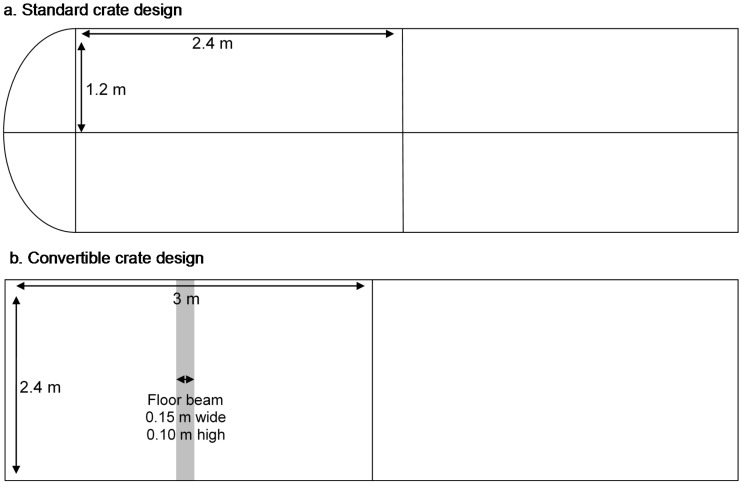
Schematic diagram of vehicle designs (**a**) standard and (**b**) convertible crates.

**Figure 2:**
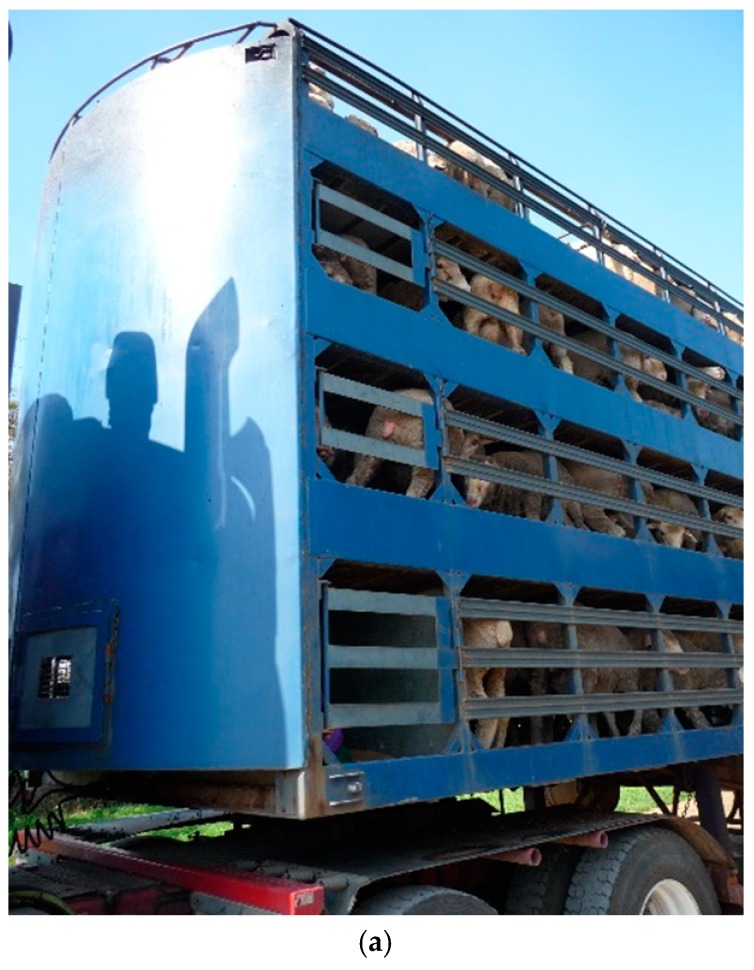

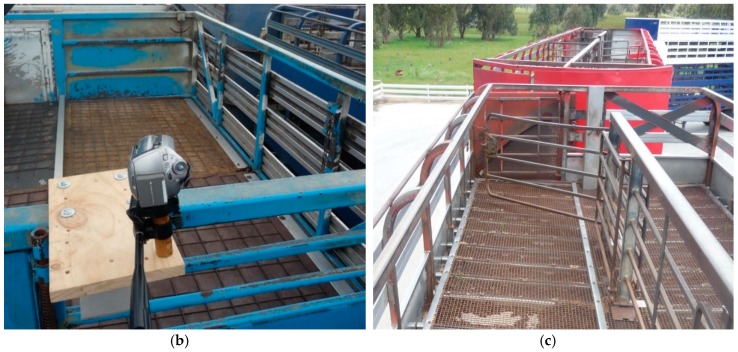
View showing (**a**) sides of a standard 4-tier commercial sheep transport vehicle, (**b**) sides of the top deck of a convertible, and (**c**) top deck of a standard crate.

**Figure 3:**
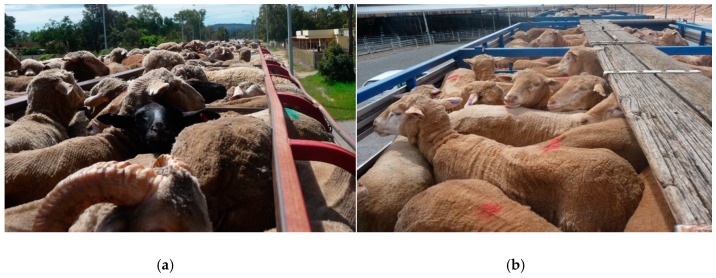
Views of merino sheep on the top deck in a (**a**) standard crate and (**b**) convertible crate. Note the curved side rail in (**a**) and straight side rail in (**b**).

The aim of this study was to examine the behavioural responses of sheep being transported in two vehicle types and to identify potential risk factors during commercial road transport. Using Qualitative Behavioural Assessment (QBA), we compared the behavioural expression of sheep transported using two types of vehicles routinely used in Australia: 1. a ‘standard’ truck design that has either a three-deck or a four-deck trailer and 2. a newer ‘convertible’ truck design with a trailer holding four decks for sheep (but which can convert to two decks for cattle) (Figure 1). Both designs have a maximum length of 27.5 m and contain some differences in the compartment or ‘crate’ in which the livestock travel. The convertible crates had slightly narrower ventilation slots and straight sides to the upper deck, whereas the standard crate had a curved side rail of the upper deck (as seen in Figure 2 and Figure 3).

The four factors examined in this study were: the design of trailer crate, the deck level of the crate, the breed of sheep, and their point of origin (reflecting immediate pre-transport handling). Differences in QBA scores for sheep between these vehicle types may be useful as an indicator of potential focal areas that require further investigation to improve livestock well-being during commercial transport.

## 2. Materials and Methods

### 2.1. Animals and Transportation

Sheep were video recorded (Figure 2) as a group during 52 short-haul (1–2 h) commercial road journeys. We filmed transport events opportunistically, and then later selected footage approximately 10 min after departure point that would allow us the opportunity to test for differences between experimental treatments (Table 1). Data was collected during routine sheep haulage over two months in Spring 2011 (Study A: Comparison of trailer crate design, breed of sheep and point of origin) and two months in Autumn 2013 (Study B: Comparison of the deck level within a crate).

The sheep filmed on all transport events were located in the fore or middle pen of the upper deck of the front crate, or the fore pen of the lower deck of commercial livestock transport vehicles registered in Western Australia. These pens were selected for their suitability for camera attachment and recording because it was unknown if adequate footage could be obtained from cameras mounted on these vehicles given the stocking densities and prevailing weather. Stocking densities and handling procedures for all transport events were at standard industry recommendations.

We carried out two studies, Study A compared the following: 1. trailer crate design, 2. sheep breed and 3. point of origin. Study B was an investigation of the effects of deck level for two trailer crate designs.

### 2.2. Study A: Comparison of 1. Trailer Crate Design, 2. Sheep Breed and 3. Point of Origin

Study A1: we compared the behavioural expression of sheep transported on the upper decks of two vehicle types: a standard crate (S) and a convertible crate (C) (Table 1; Figure 3). In the convertible crate, two decks that carry sheep are designed to fold away up against the sides of the vehicle for the cartage of cattle; hence decks 1 and 3 have fixed floors and decks 2 and 4 (upper) have non-fixed, fold-away floors. Additionally, the convertible crate has horizontal side panels rising above sheep head height, whereas the standard crate has side panels with a top side rail that curves inwards (about 250 mm from the side) above the head of the sheep, designed to prevent them from jumping out of the crate. Therefore, there is slight reduction of head room on the standard crate (Figure 3). Only sheep in the upper deck of both vehicle types were analysed in this study.

**Figure 4:**
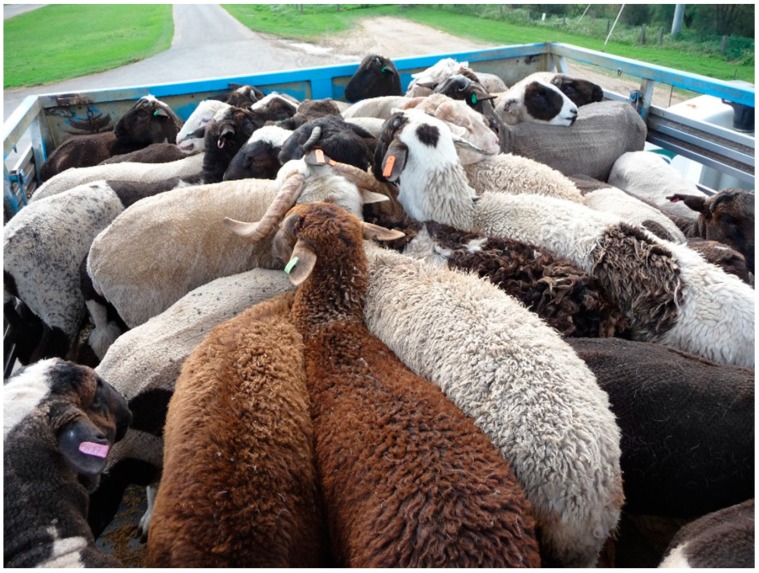
View of a typical consignment of fat-tail sheep in a convertible crate.

Study A2: we compared between merino (M) and fat-tail (F) sheep breeds. Merino groups were subjectively identified as principally merino based on physical appearance. The fat-tail group consisted of Awassi, Damara or crossbred mixes of these breeds (Figure 4). Sheep were sourced from a live export pre-assembly feedlot where they had been in the feedlot between 3 and 5 days.

Study A3: we compared merino sheep transported from two points of origin (saleyard or feedlot). Animals sourced from the saleyard had been fasted anywhere between 24 and 72 h These animals were provided with water *ad libitum* and were held in point of origin groups. The animals had been transported between 1 and 2 days before the experimental transport trip and exposed to a new environment and drafted into pens on arrival. Animals sourced for the live export feedlot had feed available up until being brought into the loading yard (2–3 h prior to the transport event). Prior to transport, sheep were held at the feedlot between 3–5 days in the same groups in which they were transported.

### 2.3. Study B: Comparison of the Deck Level within a Crate

Study B: we compared between lower (L) and upper (U) decks for Standard (S) crates (Study B1: SU-SL), lower (L) and upper (U) decks for Convertible (C) crates (Study B2: CU-CL). A final analysis included all four combinations (Study B3). The sides of the upper and lower decks for both crate designs were similar, being composed of horizontal slatted metal. The upper decks for both crate designs had an open roof. Flooring of upper decks (non-slip metal grate over a solid metal sheet) differed according to crate type; the standard crate floor was fixed and the convertible crate floor was suspended, providing slightly less stability. Pens on the lower decks had solid metal sheeting roof.

Flooring of lower decks (non-slip metal grate over a solid metal sheet) also differed according to crate type; the convertible crate had a metal beam (150 × 100 mm W × H) that traversed the front pen, providing structural support for the convertible function of the vehicle (Figure 1). This beam required the sheep to stand astride it. The size and type of the flooring grate varied slightly between vehicles, as did the amount of compacted manure that was trapped between the floor and the grate.

### 2.4. Qualitative Behavioural Assessment

#### 2.4.1. Selecting Footage for Scoring Clips

Continuous video footage was recorded during transport with a digital camera on both the upper deck (Panasonic SDR-H250, Belrose, NSW, Australia) and lower deck (GoPro Hero3 White Edition, and ContourRoam, Harvey Norman O’Connor WA, Australia) fixed to the railing of the trailer, just above sheep head height. From the footage approximately 1 min duration clips were edited out after the 10 min timepoint of the video so as not to introduce selection bias, as this was the period where the vehicle had commenced travelling at reasonable speed (on highway) and was predicted to be a time where behavioural responses of the sheep would be most marked. Clips were only rejected if the heads of the sheep were not clearly visible and subsequently taken at the next opportunity. Study A: footage was selected from approximately 10–15 min into the transport journey. Study B: clips were selected from upper and lower deck footage at the same time point at approximately 12 min after the truck departed the feedlot. Each clip was edited to highlight the focal groups by increasing the opacity of the surrounding animals in the same frame (Adobe Premiere Pro CS3 and Adobe After Effects CS3, Chatswood, NSW, Australia) and sound was removed thus obscuring evidence from the vehicle.

#### 2.4.2. Observer Demographics

Observers were recruited from University staff and students and members of the public by advertising on notice boards and email and accepting all persons that responded. Observers were offered a supermarket voucher if they attended all sessions as compensation for their time. Before participating in the scoring of sheep footage, observers were asked to complete a questionnaire regarding their demographic background, experiences with sheep, as well as ranking their attitudes and opinions towards sheep and animal welfare (Supplementary Materials S1). The majority of observers (80%) were female, most (80%) worked with animals, and half had a high level of experience with sheep (Table 2).

### 2.5. Viewing Sessions

Each observer was required to attend four sessions on campus or by correspondence (a term generation session and then three quantification sessions for each study). Observers (Study A; *n* = 26) attended sessions in November 2011 (treatment comparisons: Study A1, Study A2, and Study A3) and a second set of observers (*n* = 20; Study B) attended sessions in June 2013 (treatment comparisons: Study B1, Study B2 and Study B3). Observers were given detailed instructions on completing the QBA sessions but were not told about the experimental treatments or that the sheep were on a livestock vehicle.

For the term generation session, observers in both studies were shown 11 video clips of groups of sheep during road transport demonstrating a wide range of behavioural expression to allow observers to describe as many aspects of their expressive repertoire as possible. After watching each clip, observers were given 2 min to write down any words that they thought described that animal’s behavioural expression. There was no limit imposed to the number of descriptive terms an observer could generate, but terms needed to describe not *what* the animal was doing (i.e., physical descriptions of the animal such as vocalising, chewing, tail flicking), but *how* the animal was doing it. Subsequent editing of the descriptive terms was carried out to remove terms that described actions, and terms that were in the negative form were transformed to the positive for ease of scoring (e.g., *unhappy* became *happy*). Each descriptive term was attached to a 100 mm visual analogue scale (min = 0 to max = 100). The list of terms was alphabetically arranged, therefore effectively randomly arranged and ensuring that terms with a similar meaning were not generally listed together.

For the quantification viewing sessions, observers viewed and scored video clips of animals under transport (clips were randomly arranged within each viewing session) using their own unique list of descriptive terms. Before session commencement, observers were given detailed instructions on how to score each animal’s expression using the visual analogue scale: they were told to think of the distance between the zero-point and their mark on the scale as reflecting the intensity of the animal’s expression. Observers viewed and scored 20 clips for each quantification session except for Study A2 where they viewed and scored 10 clips and for Study B3, where they viewed and scored 36 clips. For each clip, the score was to reflect the overall expression of all sheep visible.

### 2.6. Statistical Analyses

The distance from the start of the visual analogue scale to where the observer had made a mark was measured (mm) and these measurements were entered into individual observer Excel (Microsoft Excel 2003, North Ryde, NSW, Australia) files. These data were submitted to statistical analysis with Generalised Procrustes Analysis (GPA) as part of a specialised software package written for Françoise Wemelsfelder (Genstat 2008, *VS.*N International, Hemel Hempstead, Hertfordshire, UK; Wemelsfelder et al., 2000). Each Study (treatment comparison) was analysed separately, making a total of six independent analyses.

For a detailed description of GPA procedures, see Wemelsfelder et al. [12]. Briefly summarised, GPA calculates a consensus or ‘best fit’ profile between observer assessments through complex pattern matching. GPA provides a statistic (the Procrustes Statistic) which indicates the level of consensus (i.e., the percentage of variation explained between observers) that was achieved. The statistical process whereby this best-fit pattern, termed the consensus profile, is identified takes place independently of the meaning of individual terms used by observers. Whether this consensus is a significant feature of the data set, or, alternatively, an artefact of the Procrustean calculation procedures, is determined through a randomisation test [23]. This procedure rearranges at random each observer’s scores and produces new permutated data matrices. By applying GPA to these permutated matrices, a ‘randomised’ profile is calculated. This procedure is repeated 100 times, providing a distribution of the Procrustes Statistic indicating how likely it is to find an observer consensus based on chance alone. Subsequently a one-way *t*-test is used to determine whether the actual observer consensus profile falls significantly outside the distribution of randomised profiles.

Through Principle Components Analysis (PCA), the number of dimensions of the consensus profile is reduced to several main dimensions (usually 2 or 3) explaining the variation between animals. GPA dimensions are interpreted by correlating the animals’ scores to the observers’ individual scoring patterns, producing individual observer word charts that describe the consensus dimensions through their association with each individual observer’s terms. These word charts can then be compared for linguistic consistency. From these word charts, a list of terms describing the consensus dimensions was produced, by selecting terms for each observer that correlated strongly with those dimensions (i.e., the more highly correlated an individual term is with a dimension end, the more weight it has as a descriptor—positive or negative—for that dimension). Each video clip of animals receives a quantitative score on each of these dimensions, so that the transport trip’s position in the consensus profile can be graphically represented in two- or three-dimensional plots. Each plot represents each of the transport trips in the respective treatments, where the position of the transport trip indicates its scores on each GPA axis.

To investigate treatment effects, the observer scores for each GPA dimension (response variables) were analysed using mixed-model ANOVAs, with driver ID and observer ID included as random factors to account for driver behaviour/truck design differences and repeated observations. For Study B3, we included both crate and deck as two independent factors. Analyses were carried out using Statistica software, version 9 (StatSoft-Inc, Tulsa, OK, USA).

### 2.7. Survey of Drivers

To gain preliminary information about drivers and procedures involved with commercial livestock carriers, two short surveys were carried out while we were collecting footage (Spring 2011). Each driver was given a questionnaire (Supplementary Materials S2) to determine driver background and their opinions of the livestock transport industry. Information on each journey was gathered using a questionnaire that was completed both through viewing the loading process and questioning the driver (Supplementary Materials S3).

## 3. Results

**Figure 5:**
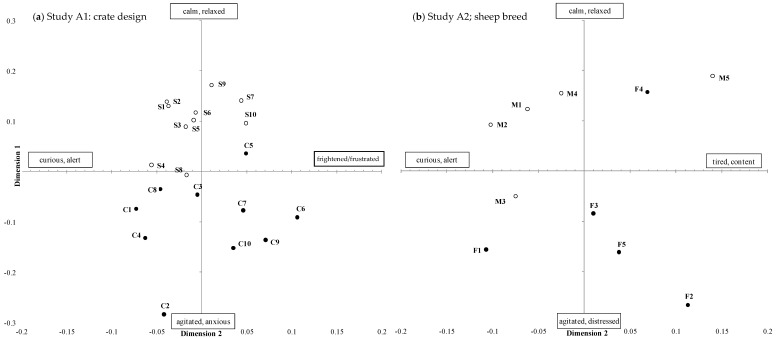
Positions of individual transport clips (represented by numbers) on Generalised Procrustes Analysis (GPA) dimensions 1 (y axes) and 2 (x axes). (**a**) Study A1: crate design experiment (S: standard crate, open circles, or C: convertible crate, closed circles); (**b**) Study A2: sheep type experiment (M: Merino sheep, open circles, or F: Fat-tail sheep, closed circles). Bold text indicates GPA dimensions 1 for both studies were statistically significantly different between treatments.

**Figure 6:**
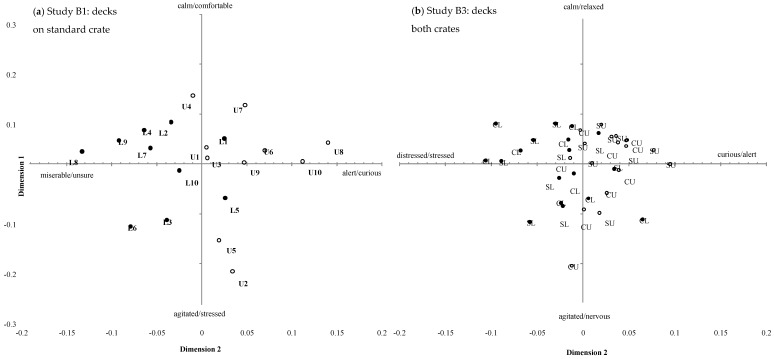
Positions of individual transport clips on GPA dimensions 1 and 2 obtained for (**a**) Study B1 standard crate experiment (Numbers represent each transport trip, U: upper deck, open circles, or L: lower deck, closed circles) and (**b**) Study B3 crate design experiment (SU: standard crate upper deck, open circles, SL: standard crate lower deck, closed circles, CU: convertible crate upper deck, open circles and CL: convertible crate lower deck, closed circles). Bold text indicates GPA dimensions 2 for both studies were statistically significantly different between treatments.

**Figure 7:**
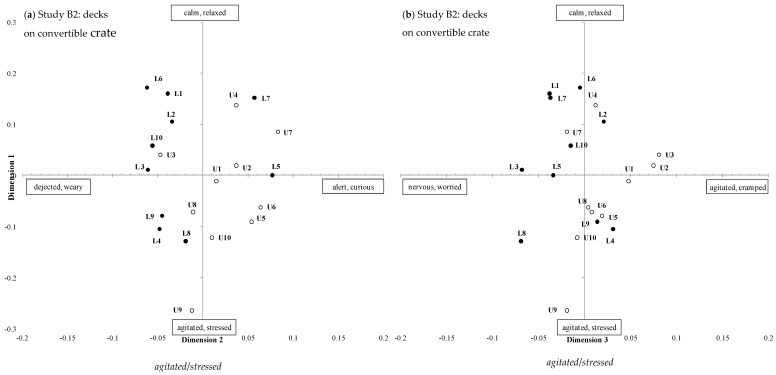
Positions of individual transport clips (numbers represent each transport trip) obtained from Qualitative Behavioural Assessment in Study B2 for convertible crate experiment (U: upper deck, open circles, or L: lower deck, closed circles) showing the same data plotted for (**a**) GPA dimensions 1 and 2 and (**b**) GPA dimensions 1 and 3.

The 26 observers participating in Study A generated a total of 115 unique terms to describe the behavioural expression of the sheep viewed in the term generation session, with an average of 17 ± 6 (min: 6, max: 31) terms per observer. The 20 observers participating in study B generated a total of 85 unique terms, with an average of 12 ± 4 (min: 6, max: 21) terms per observer. There was significant consensus between observer scores for each of the six treatment comparisons; Procrustes Statistics are shown in Table 3. Terms with the strongest correlation with each of the GPA dimensions for each treatment comparison are shown in Table 3 (max 10 terms); the first two terms are used as labels for each GPA dimension (Figure 5, Figure 6 and Figure 7).

We recorded significant effects of vehicle driver (included as a random factor in all our analyses) on GPA dimension scores for all except Study B3 (Table 3).

### 3.1. Study A: Comparison of 1. Trailer Crate Design, 2. Sheep Breed and 3. Point of Origin

#### 3.1.1. Study A1 Trailer Crate Design: Standard Crate vs. Convertible Crate

The first three GPA dimensions explained a total of 66.5% of the variation between animals (Table 3). The positions of individual sheep on the first two GPA dimensions are shown in Figure 5a. There was a significant treatment effect for GPA dimension 1 (*p* < 0.001) with sheep transported in a standard crate scored as more *calm*/*relaxed* than sheep transported in a convertible crate, which were scored as more *agitated*/*anxious*. There were no significant treatment effects on GPA dimensions 2 or 3. Of the transport trips assessed, 19 of them stocked adult sheep. One transport journey that stocked lambs; this transport journey is labelled ‘C2’ in Figure 5a and stood out as an outlier, with sheep in this clip scored as more agitated/anxious than any other clip.

#### 3.1.2. Study A2 Sheep Breed: Merino vs. Fat-Tail Sheep

The first three GPA dimensions explained a total of 65.0% of the variation between animals (Table 3). The positions of individual sheep on the first two GPA dimensions are shown in Figure 5b. There was a significant treatment effect on GPA dimensions 1 (*p* = 0.006) and 2 (*p* = 0.011) with fat-tail sheep scored as more *agitated*/*distressed* (GPA1) and *curious*/*alert* (GPA2) than merino sheep, which were scored as more *calm*/*relaxed* and *tired*/*content*. There were no significant treatment effects on GPA dimension 3.

#### 3.1.3. Study A3 Point of Origin: Feedlot vs. Saleyard

The first three GPA dimensions explained a total of 63.1% of the variation between animals (Table 3). There was a significant treatment effect on GPA dimension 3 (*p* = 0.014), with sheep transported from the feedlot scored as more *comfortable*/*relaxed* than sheep transported from the saleyard, which were more *nervous*/*curious*. However this dimension only explained a small proportion (5.8%) of the variability in data. There were no significant differences in GPA dimension 1 and 2 scores.

### 3.2. Study B: Comparison of the Deck Level within a Crate

#### 3.2.1. Study B1 Deck Level: Standard Crate Upper vs. Lower Deck

The first three GPA dimensions explained a total of 67.6% of the variation between animals (Table 3). The positions of individual sheep on the first two GPA dimensions are shown in Figure 6a. There was a significant treatment effect for GPA dimension 2 (*p* < 0.001) and 3 (*p* < 0.001), with sheep transported in the upper deck scored as significantly more *alert*/*curious* and *stressed*/*alert* than sheep transported in the lower deck, which were scored as more *miserable*/*unsure* and *nervous*/*agitated*. There were no significant treatment effects on GPA dimension 1.

#### 3.2.2. Study B2 Deck Level: Convertible Crate Upper vs. Lower Deck

The first three GPA dimensions explained a total of 72.4% of the variation between animals (Table 3). The positions of individual sheep on the three GPA dimensions are shown in Figure 7a,b. There were significant treatment effects for all three GPA dimensions, with sheep on the upper deck scored as significantly more *calm*/*relaxed*, *alert*/*curious*, and *agitated*, *cramped* than sheep on the lower deck, which were scored as more *agitated*/*stressed*, *dejected*/*weary* and *nervous*/*worried*.

#### 3.2.3. Study B3 Standard versus Convertible Crate: Upper and Lower Deck

The first three GPA dimensions explained a total of 71.1% of the variation between animals (Table 3). The positions of individual sheep on the first two GPA dimensions are shown in in Figure 6b. There was no significant effect of crate on the GPA dimension scores. There was a significant effect of deck for GPA dimension 2 (*p* = 0.008) with sheep on the upper decks during road transport scored as more *curious*/*alert* than sheep on the lower decks, which were scored as more *distressed*/*stressed*. The crate x deck interaction terms were not significant.

### 3.3. Driver Questionnaire

Fourteen drivers that participated in the study were asked what they believed should be the most important priorities for improvement of the livestock transport industry. Their responses were;

- Five drivers believed that the industry required better enforcement of withholding (curfew) periods.
- Drivers expressed concern about the waiting time at the wharf prior to sheep being loaded for export.
- Three drivers believed that loading density is an important priority; in particular, the density should be slightly lower, especially on long distance trips.
- Two drivers stated that education was required for producers to better prepare animals for transport and therefore improve loading and reduce transport stress and to understand the benefits of lower densities. Such preparation could include habituation to loading ramps, handling and humans, and education on the design of loading facilities and minimum requirements (e.g., a raised loading ramp).
- Two drivers identified that they required better support in refusing to load animals that they believed were in poor condition or not fit to load.

In response to being asked what drivers believed were the most important animal welfare issues that needed to be addressed during road transport, the following was indicated:

- condition of animal and impact of condition on stress and welfare during transport;
- having the right to refuse an emaciated or pregnant animal, guidelines for stocking rate during transport;
- rest yards at east-west state border with feed and water available; and
- driver education for handling sheep during loading and unloading.

In response to being asked what specific behaviour drivers thought would indicate that a sheep’s welfare was compromised during transport, the following was indicated:

- body position: lying down, in corner of crate or underneath other sheep, legs out of crate;
- movements such as limping or tucked up when loading or unloading;
- breathing style: reparation distress, tongue out when panting;
- general demeanour: head down, position of eye; and
- vocalisation.

## 4. Discussion

Using Qualitative Behavioural Assessment, observers from a range of backgrounds and with different levels of sheep experience distinguished behavioural expression of sheep subjected to different physical conditions during commercial transport (e.g., trailer crate type and deck level), between sheep of different breeds and even between sheep transported from a livestock saleyard compared with those from a feedlot. Our previous studies under experimental transport conditions showed behavioural expression reflected changes in the underlying physiological state of the livestock that were typically associated with the stress response [15,16,17,18]. Such behavioural responses are likely to predict the wellbeing of the animals, and as indicators of welfare they may be associated with production traits [24,25]. Importantly, the early identification of animal responses to husbandry procedures (e.g., farm/facility/transport setting) allows the modification of procedures or facilities in a way that optimises the health and welfare of animals [26,27]. Even subtle differences in demeanour can therefore be informative.

### 4.1. Study A1: Trailer Crate Design

The observers detected differences in merino sheep travelling in the upper deck of two types of trailer crates over the same road journey. Sheep in the convertible crate were scored as more *agitated*/*anxious* than sheep in the standard crate, which were more *calm*/*relaxed*. Hence, this dimension corresponds to a distinction between emotional valence of sheep, with similar terms used to those in our previous studies using a small, single-deck trailer [15,16]. Prior to this experiment, sheep had been handled in a similar way; they were yarded 2–3 h before loading, had minimal food curfew and were moved through the same loading facility.

Although the two crate designs had similar loading density, the larger pen size in the convertible crate allowed the animals the freedom to pack tightly together in the corners, leaving others more room to move. This may have allowed more physical movement and less sheep-to-sheep support within the whole pen; increased space availability has been demonstrated to increase movement in lambs, but also increase slipping and carcass bruising [28,29]. Structural differences in the sides and the floor of the upper decks of the two crates may have influenced how sheep travelled. The slight reduction of head room caused by the side rails on the standard crate may decrease physical movement or create a more confined feeling for the sheep. The floor of the upper deck of the convertible crate is less stable as it allows greater suspension and vibration than that of the standard crate. One possible explanation is that this increased flexibility of the floor may lead to sheep experiencing less secure footing.

### 4.2. Study A2: Sheep Breed

Observers detected differences in merino and fat-tail sheep travelling in the same type of trailer crate (a standard crate) over the same road journey. Merino sheep were scored as more *calm*/*relaxed* and *curious*/*alert* compared with the Fat-tail sheep on the same vehicle and route, which were scored as more *agitated*/*distressed* and *tired*/*content*. These dimensions appear to reflect activity and/or arousal. However, given that we only had five replicate transport events for each sheep breed group, our data warrants following up with additional studies on how different sheep breeds deal with road transport.

Prior to this experiment, sheep had been handled in a similar way (yarded 2–3 h earlier, with no feed curfew and moved through the same loading facility). Thus, the behavioural response to transport appeared to be linked to breed. The behavioural responses may reflect the ability of the sheep to cope with the transport stressors and this may be linked to their state of fearfulness. Previous studies have demonstrated physiological differences (plasma cortisol, packed cell volume) in sheep genotypes, with some more reactive to land transport than others [30]. In a non-transport example, Miller et al. [31] demonstrated that QBA scores were different for feral goats exposed to varying levels of human interaction, suggesting that the scores reflected habituation and possibly their ability to adapt to the challenge of confinement. Grandin [32] found that animals with an excitable temperament may have greater difficulty adapting to a situation, while calmer animals may adapt more easily and become less stressed. Identification and selection of breeds of sheep that better cope with the transport environment represents one way to improve the welfare of livestock.

### 4.3. Study A3: Point of Origin

We only found subtle differences in the demeanour of sheep that originated from either a saleyard or feedlot—the only differences were on GPA dimension 3, which only accounted for 5.8% of the total variation in observer scores. Merino sheep being transported from a saleyard were more *nervous*/*curious* than those transported from a feedlot, which were more *comfortable*/*relaxed*. We had expected that there would be differences in demeanour for these animals because sheep had been exposed to very different experiences over the preceding 24–48 h. Prior to yarding for 2–3 h before loading, sheep from the feedlot had been fed in a pre-export feedlot for approximately 5 days. These sheep were penned in large flocks and most likely habituated to the feed, shed and daily routine; they also had minimal time off food and water prior to transport. By contrast, sheep loaded at the saleyard had experienced a novel, noisy and high arousal environment over the previous 1–3 days. Potential stressors for saleyard sheep include a transport journey from their farm of origin, feed curfew of 24–72 h, separation and mixing of social groups, handling through several small yards by sheep dogs, as well being confronted by the presence of humans at close proximity. The subtle differences in demeanour could indicate that the transport environment is sufficiently novel and challenging to mostly overshadow prior affective condition. Further studies comparing more diverse backgrounds, such as from farm of origin and comparing varied lengths of time in confinement or curfews could provide further insight into the effect of point of origin.

### 4.4. Study B: Deck Level

We recorded a significant difference in the demeanour of sheep positioned in the upper and lower decks on for both vehicle crate types, with sheep on the upper deck scored as more *alert*/*curious* and *agitated*/*cramped* than those on the lower deck, which were scored as more *miserable*/*unsure*, *dejected*/*weary*, *nervous*/*worried* and *distressed*/*stressed*. The final study that allowed observers to compare sheep expression directly from journeys from all four positions, that is standard upper and lower and convertible upper and lower, confirmed the significant difference was detected between deck levels but not crate type, indicating that effect of deck level overshadowed any difference between crate design. Interestingly, the term *nervous* was highly loaded on the low arm of the GPA3 for all but one analyses but the term *cramped* was only loaded on GPA3 analysis of CU-CL. Although the third dimension accounts for a small percentage of variation, it seems sheep in the upper deck were viewed as more cramped than the lower.

How important this deck position is to sheep is unknown and may be influenced by other factors such as weather and climate. Sheep positioned on the upper deck are likely to have more visual and tactile stimulation as they experience wind, sun or rain, which may affect how they experience the journey whereas those in lower decks are largely screened from such exposure. It has been demonstrated that many novel aspects associated with transportation, apart from the vehicle movement itself, can trigger behavioural responses in sheep [33]. The behavioural dimensions identified in this study concur with key qualitative descriptors for sheep demeanor that were identified in previous studies, such as *agitated*, *distressed*, *alert* and *curious* [13,15,16,34]. These dimensions used terms semantically consistent with a valence of ‘mood’ (GPA 1) and ‘arousal’ (GPA2) that have been similarly reported elsewhere [13,27,31,34].

### 4.5. Limitations of This Study

Because we were working under commercial conditions and made every attempt to not interfere with normal transport processes, we had relatively little control over the conditions that animals were transported under. For example, we had only few transport events for fat-tail sheep that were transported under matched conditions as merino sheep, which restricted our sample size for this treatment comparison. Furthermore, in order to restrict the number of variables between transport events, clips were taken from routine transport events that matched each other in as many ways as possible.

The convertible crate design is relatively new, and only a few livestock trucks of such design in Western Australia. Multiple transport events on specific vehicles (driven by its owner/driver) were therefore not strictly independent. We tested for such an effect in our data (driver included as a random factor) and identified significant effects of vehicle driver on the demeanour of sheep, which could reflect either their driving style or the specific design of their vehicle.

Finally, although we attempted to obscure evidence of the animals being on a truck by editing our video clips, we cannot guarantee that observers were unaware of the treatment difference for our deck level experiment. Obtaining footage from the lower decks with adequate lighting and adequate visualisation of groups of sheep with limited head room was challenging. The contrast with sheep in sunshine on the upper deck could not be avoided. Such cues are likely to influence how observers score footage because QBA scoring is sensitive to environmental clues which can introduce undesirable bias due to the observers’ judgment of that context [35,36]. We used different cameras to account for different lighting levels on the different decks and therefore the quality of the footage was equitable, but animals on the lower decks are more obviously in a confined place than those on the top deck.

### 4.6. Driver Questionnaire

We found significant effects of driver ID (included as a random factor in our analyses) on the GPA dimension scores (for all but Study B3), which suggested that individual driver behaviour or truck design influenced the behaviour of independent groups of sheep transported in their trucks. Cockram et al. [6] found a significant effect of driver (comparing two drivers) on losses of balance and disturbance in sheep transported by truck, with ~80% of losses of balance caused by driving events such as acceleration, braking, cornering and uneven road surfaces. Awareness of driving style can help improve driving style to minimize stress to the animals, and Grandin [20,37] showed that there was an increase in the welfare of animals when livestock drivers received a bonus payment for meat quality of the animals transported. On the other hand, truck design is also likely to influence animal welfare during transport [38]. For example, Huertas et al. [29] found differences in carcass bruising with different truck designs. We could not distinguish between driver and truck in our study—this would require an experimental design where multiple drivers used the same truck which was not within the scope of our study.

The short driver survey allowed researchers to informally discuss the concerns of a small number of drivers directly responsible for the care of transported animals. Some areas for improvement identified, including handling, preparation of sheep before transport (length of curfew) and long-distance journeys. Importantly, drivers were able to describe sheep behaviour that alerted them to situations of poor animal welfare. These surveys allowed the initiation of dialogue about the shared responsibility of ensuring livestock were well cared for during land transport. A willingness to participate in future research and monitoring was a useful outcome from such conversations. Further engagement of industry at all levels with research is required to ensure continued positive changes to livestock transport. Importantly, the drivers did not volunteer driving style or truck design as an issue of welfare concern. In informal discussions with individual drivers, they raised their observations about differences between deck levels, although they did not identify this as an animal welfare issue in their formal surveys.

## 5. Conclusions

Observers showed strong agreement as to how the sheep were rated, supporting the use of human observers to score sheep behaviour during commercial transport, with minimum intrusion into the normal procedures of animal handling. QBA is a valuable method for assessing the wellbeing of sheep in production systems [14]. Observer rating of animal behaviour offers many advantages, not the least that it is practical and relatively inexpensive. The method is also repeatable, reliable and relevant to animal welfare assessment.

The livestock transport process is complex and involves handling of animals at multiple facilities by different groups of stockpersons working under time constraints. Hence a non-invasive assessment using video recording may prove an efficient and effective means to measure the animals’ responses to handling and movement and offers insight into how well livestock are coping in what are stressful, but necessary, environments. The inclusion of a qualitative measure adds an important interpretative element to any welfare analysis or assurance scheme. With further validation at specific points within the livestock production chain, QBA appears as a promising welfare-relevant monitoring tool for industry.

## Figures and Tables

**Table 1:** Description of the treatment comparisons carried out. Bold text indicates the comparison made for each study.

Study	Vehicle Cate ^†^ S: Standard, C: Convertible	Deck U: Upper, L: Lower	Sheep M: Merino, F: Fat-Tail	Origin ^‡^	Number of Video Clips Viewed by Observers
A1 Vehicle crate	**S vs. C**	U	M	FL	*n* = 10, 10
A2 Sheep breed	S	U	**M vs. F**	FL	*n* = 5, 5
A3 Point of origin	S	U	M	**SY vs. FL**	*n* = 10, 10
B Deck level:					
B1 (SU-SL)	S	**U vs. L**	M	FL–W	*n* = 10, 10
B2 (CU-CL)	C	**U vs. L**	M	FL–W	*n* = 10, 10
B3 (SU-SL-CU-CL)	**S vs. C**	**U vs. L**	M	FL–W	*n* = 18, 18

^†^: Vehicles with the standard crate design had four 1.2 × 2.4 m W × L (2.9 m^2^) pens per deck and a solid metal floor that was fixed in place. Vehicles with the convertible crate design had two 2.4 × 3.0 m W × L (7.2 m^2^) pens per deck and a solid metal floor that was not permanently fixed in place. ^‡^: The routes taken included a period of continuous and stop-start driving, originating from either SY: saleyard (Muchea, Western Australia) to feedlot, or FL: from a registered premises feedlot (Baldivis, Western Australia) to a loading wharf (Fremantle, Western Australia).

**Table 2:** Demographic description of observers that contributed to QBA scoring.

Attribute	Category: # of Observers	
Study A (26 observers)		
Sex	Female: 21	Male: 5
Country of birth	Australia: 15	Other: 11
Residence	Urban: 24	Rural: 2
Area of study/employment: animal-related	Yes: 24	No: 2
Dietary preference: vegetarian	Yes: 4	No: 22
Purchasing habit: purchases own meat/eggs/dairy	Yes: 25	No: 1
Pet ownership	Yes: 22	No: 4
Level of experience with sheep	Low: 7; Medium: 4; High: 15	
Age (years)	<19: 1 20–29: 15 30–39: 8 40–49: 1 50–59: 0 60–69: 1 >70: 0	
Study B (26 observers)		
Sex	Female: 16	Male: 4
Country of birth	Australia: 11	Other: 9
Residence	Urban: 16	Rural: 4
Area of study/employment: animal-related	Yes: 13	No: 7
Dietary preference: vegetarian	Yes: 4	No: 16
Purchasing habit: purchases own meat/eggs/dairy	Yes: 20	No: 0
Pet ownership	Yes: 19	No: 1
Level of experience with sheep	Low: 7 Medium: 5 High: 8	
Age (years)	<19: 0 20–29: 8 30–39: 7 40–49: 2 50–59: 2 60–69: 0 >70: 1	

**Table 3:** Terms used by observers to describe sheep behavioural expression during road transport. Terms showing the strongest correlations (*r* > 0.5) with low and high values on each Generalised Procrustes Analysis (GPA) dimension of the consensus profile (maximum 10 terms presented). Order of terms is determined firstly by number of observers to use that term (in parentheses where >1) and secondly by weighting of each term.

Treatment, Procrustes Statistic ^†^	GPA Dimension	Low Values	High Values	Treatment Effect
Study A				
A1: S-C PS: 56.64% (*t*_99_ = 43.8, *p* < 0.001)	1 (53.2%)	Agitated (7), anxious (5), nervous (5), worried (5), concerned (3), scared (3), jumpy (2), fidgety (2), alert (2), distressed (2)	Calm (11), relaxed (7), settled (3), restful (2), comfortable (2), subdued, happy, tolerant, accepting, bored	Crate: F_1, 6_ = 48.85, *p* < 0.001 Sheep in a standard crate scored higher than sheep in a convertible crate Driver ID (random factor): F_6, 487_ = 5.90, *p* < 0.001
	2 (8.5%)	Curious (7), alert (3), inquisitive (2), nervous (2), interested, confident, comfortable, puzzled, at ease, watchful	Frightened, frustrated, agitated, bored, annoyed, distressed, anxious, bossy	Crate: F_1, 6_ = 0.29, *p* = 0.609 Driver ID (random factor): F_6, 487_ = 26.84, *p* < 0.001
	3 (4.8%)	Tired (2), apprehensive, struggling, pissed off, sad, fidgety, certain, alert, sleepy, bored	Confident, relaxed, sleepy, depressed, settled, satisfied	Crate: F_1, 6_ = 0.96, *p* = 0.367 Driver ID (random factor): F_6, 487_ = 6.67, *p* < 0.001
A2: M-FT PS: 56.11% (*t*_99_ = 12.01, *p* < 0.001)	1 (42.5%)	Agitated (6), distressed (6), nervous (5), anxious (4), jumpy (3), pushy (2), alert (2), wary (2), frustrated (2), fidgety (2)	Calm (6), relaxed (4), comfortable (4), happy (3), settled (2), patient (2), composed (2), restful, mellow, unphased	Breed: F_1, 4_ = 27.98, *p* = 0.006 Merino sheep scored higher than fat-tail sheep Driver ID (random factor): F_2, 223_ = 30.87, *p* < 0.001
	2 (12.6%)	Curious (5), alert (4), inquisitive (4), worried (3), interested (2), comfortable (2), nervous (2), attentive, aware, confident	Tired (2), content (2), happy, calm, scared, settled, annoyed, anxious, frustrated, nervous	Breed: F_1, 14_ = 8.44, *p* = 0.011 Merino sheep scored lower than fat-tail sheep Driver ID (random factor): F_2, 223_ = 7.30, *p* = 0.001
	3 (9.9%)	Nervous (3), alert (3), interested (2), curious (2), annoyed (2), calm (2), jumpy, nonchalant, anticipating, restless	Relaxed (3), tired (2), alert (2), anxious (2), scared (2), comfortable (2), sleepy (2), unsure, insecure, resigned	Breed: F_1, 3_ = 0.05, *p* = 0.837 Driver ID (random factor): F_2, 223_ = 75.96, *p* < 0.001
A3: FL-SY PS: 49.2% (*t*_99_ = 29.051, *p* < 0.001)	1 (46.6%)	Agitated (4), anxious (4), frightened (3), worried (3), nervous (3), panicked (3), scared (2), restless (2), jumpy, fretful	Calm (6), bored (3), relaxed, casual, accepting, placid, comfortable, settled, patient, composed	Origin: F_1, 6_ = 0.12, *p* = 0.745 Driver ID (random factor): F_5, 469_=27.51, *p* < 0.001
	2 (10.7%)	Curious (3), happy (2), alert (2), bored (2), interested (2), comfortable, calm, settled, resigned, content	Curious (2), agitated (2), resigned, interested, tired, lethargic, depressed, struggling, distressed, bored	Origin: F_1, 18_ = 1.12, *p* = 0.304 Driver ID (random factor): F_5, 469_=4.21, *p* < 0.001
	3 (5.8%)	Nervous (2), curious (2), distressed, excited, interested, agitated, annoyed, alert	Comfortable (2), relaxed (2), aware, stressed, mellow, calm, settled, confident, happy, agitated	Origin: F_1, 24_ = 7.04, *p* = 0.014 Sheep from feedlot scored higher than sheep from saleyard Driver ID (random factor): F_5, 469_ = 3.11, *p* = 0.009
Study B				
B1: SU-SL PS: 42.75% (*t*_99_ = 16.45, *p* < 0.001)	1 (35.9%)	Agitated (7), stressed (4), pushy (3), distressed (3), worried (3), uneasy (2), restless (2), nervous (2), scared(2), distressed (2)	Calm (7), comfortable (4), relaxed (2), happy (2), placid, quiet, accepting	Deck (S): F_1, 377_ = 0.41, *p* = 0.521 Driver ID (random factor): F_2, 377_ = 15.03, *p* < 0.001
	2 (20.9%)	Miserable, unsure, tired, restful	Alert (9), curious (6), interested (5), happy (3), inquisitive (2), watchful (2), comfortable (2), observant, confused, confident	Deck (S): F_1, 377_ = 136.22, *p* < 0.001 Sheep transported in the upper deck scored higher than sheep transported in the lower deck Driver ID (random factor): F_2, 377_ = 6.04, *p* = 0.003
	3 (10.8%)	Nervous (2), agitated (2), stressed, distressed, settled, weary, wary, dejected, collected, confused	Stressed (2), alert (2), restless (2), distressed, adjusting, anxious, quiet, fidgety, unsupported, relaxed	Deck (S): F_1, 377_ = 25.40, *p* < 0.001 Sheep transported in the upper deck scored higher than sheep transported in the lower deck Driver ID (random factor): F_2, 377_ = 4.21, *p* = 0.015
B2: CU-CL PS: 45.34% (*t*_99_ = 23.67, *p* < 0.001)	1 (56.1%)	Agitated (6), stressed (5), nervous (4), frightened (4), restless (4), distressed (3), uneasy (3), anxious (2), unsettled (2), worried (2)	Calm (9), relaxed (7), comfortable (6), happy (3), quiet (2), resolved, settled, at ease, resigned, restful	Deck (C): F_1, 376_ = 22.54, *p* < 0.001 Sheep transported in the upper deck scored higher than sheep transported in the lower deck Driver ID (random factor): F_3, 376_ = 32.58, *p* < 0.001
	2 (8.5%)	Dejected, weary, agitated, tired, scared	Alert (6), curious (4), interested (2), confident (2), relaxed (2), happy, wary, observant, aware	Deck (C): F_1, 376_ = 22.54, *p* < 0.001 Sheep transported in the upper deck scored higher than sheep transported in the lower deck Driver ID (random factor): F_3, 376_ = 32.58, *p* < 0.001
	3 (7.8%)	Nervous, worried, unsure	Agitated (2), cramped (2), curious (2), alert, aware, interested, relaxed, squashed	Deck (C): F_1, 376_ = 49.17, *p* < 0.001 Sheep transported in the upper deck scored higher than sheep transported in the lower deck Driver ID (random factor): F_3, 376_ = 24.54, *p* < 0.001
B3: SU-SL-CU-CL PS: 36.86% (*t*_99_ = 32.699, *p* < 0.001)	1 (43.2%)	Agitated (5), nervous (3), restless (3), uneasy (3), frightened (3), anxious (2), stressed (2), worried (2), distressed (2), confused (2)	Calm (7), relaxed (5), comfortable (4), happy, at ease, settled	Crate: F_1, 5_ =0.35, *p* = 0.581 Deck: F_1, 6_ = 0.01, *p* = 0.968 Crate x deck: F_1, 5_ = 0.63, *p* = 0.466 Driver ID (random factor): F_5, 5_ = 4.30, *p* = 0.065
	2 (19.1%)	Distressed (2), stressed (2), bored, scared, freaked, miserable, quiet, unsure, worried, sad	Curious (8), alert (7), aware (3), interested (2), observant (2), confident, happy, relaxed	Crate: F_1, 6_ = 0.47, *p* = 0.525 Deck: F_1, 8_ = 12.35, *p* = 0.008 Sheep transported in the upper deck scored higher than sheep transported in the lower deck Crate x deck: F_1, 5_ = 0.51, *p* = 0.509 Driver ID (random factor): F_5, 5_ = 1.42, *p* = 0.355
	3 (8.8%)	Nervous (2), curious (2), distressed, excited, interested, agitated, annoyed, alert	Quiet (2), collected, calm, confused, accepting, aloof, peaceful	Crate: F_1, 5_ < 0.01, *p* = 0.994 Deck: F_1, 4_ = 4.50, *p* = 0.114 Crate x deck: F_1, 4_ = 0.82, *p* = 0.412 Driver ID (random factor): F_5, 5_ = 2.02, *p* = 0.222

^†^ PS: Procrustes Statistic showing the percentage of variance in the dataset that could be attributed to the consensus in scoring between individual observers and the results for the one-way *t*-test comparing this result with a mean randomised profile of the same dataset, indicating that the consensus between observers in their use of descriptive terms to quantify the behavioural expression of these sheep was statistically significant.

## Supplementary Materials

The following are available online at http://www.mdpi.com/2076-2615/8/11/209/s1.
