# Supplementary material for: Qualitative Behavioural Assessment as a Method to Identify Potential Stressors during Commercial Sheep Transport
Source: Animals (Basel). 2018 Nov 15;8(11):209. doi: 10.3390/ani8110209 (PMC6262568; doi:10.3390/ani8110209)
Supplement: Supplementary file 1 [file animals-08-00209-s001.pdf]

Supplementary Materials: Qualitative Behavioural Assessment as a Method to Identify Potential Stressors During Commercial Sheep Transport

Teresa Collins [1,*], Catherine A. Stockman [1], Anne L. Barnes [1], David W. Miller [1], Sarah L. Wickham [1] and Patricia A. Fleming [1*]

School of Veterinary & Life Sciences, Murdoch University, Murdoch, WA 6150, Australia; c.stockman@murdoch.edu.au (C.A.S.); a.barnes@murdoch.edu.au (A.L.B.); D.Miller@murdoch.edu.au (D.W.M.); sarahlwickham@outlook.com (S.L.W.); t.fleming@murdoch.edu.au (P.A.F.)

* Correspondence: t.collins@murdoch.edu.au;

Received: 3 October 2018; Accepted: 9 November 2018; Published: 15November 2018

Supplementary Materials S1: Section 1 of Questionnaire.

To keep your identity anonymous please create a unique code from the following

First two letters of your mothers first name ☐☐

First two letters of your fathers first name ☐☐

Day of the month you were born (e.g. 4th = 04, 17th = 17) ☐☐

These four letters and two numbers make up your unique code (e.g. SIAL17)

Your Unique Code is: ____________________________

1. Age: ☐ 19 or under ☐ 20–29 ☐ 30–39 ☐ 40–49 ☐ 50–59 ☐ 60–69 ☐ 70+
2. Gender: ☐ Male ☐ Female
3. Country of birth
4. Nationality
5. Current position ☐ Undergraduate student ☐ Postgraduate student ☐ Staff ☐ Other

6. Area of study/employment ☐ Biological Science & Biotechnology ☐ Business ☐ Chemical & Mathematical Science ☐ Chiropractic ☐ Engineering & Energy ☐ Environmental Science ☐ Education ☐ IT ☐ Law ☐ Media ☐ Nursing ☐ Pharmacy ☐ Psychology ☐ Sustainability

☐ Social Science & Humanities ☐ Animal Science ☐ Veterinary science ☐ Biomedical Science ☐ Other

7. Do you **currently** live on: ☐ an urban property/environment
☐ a rural property/environment

8. Have you **ever lived** on a rural property/environment ☐ Yes ☐ No
If yes, how long did you live there

☐ Less than 1 month

☐ 1 – 6 months

☐ 6 – 12 months

☐ 1 – 2 years

☐ More than 2 years

9. Have you ever **visited a farm** which rears animals? ☐ Yes ☐ No
If yes, what animals did they farm (tick all that apply)

- ☐ Sheep (wool)
- ☐ Sheep (meat)
- ☐ Beef cattle
- ☐ Dairy cattle
- ☐ Chickens (eggs)
- ☐ Chickens (meat)
- ☐ Pigs

Other

10. Have you ever **visited an abattoir**? ☐ Yes ☐ No
If yes, what type of animal was being slaughtered there

☐ Sheep ☐ Cattle ☐ Pig ☐ Chicken

Other

11. Have you ever **visited a livestock saleyard**? ☐ Yes ☐ No
If yes, what type of animal was being sold there:

☐ Sheep ☐ Cattle

Other

12. How often do you see sheep being transported?
- ☐ Daily
- ☐ Once or twice a week
- ☐ Once a fortnight
- ☐ Once a month
- ☐ Once a year
- ☐ A few times a year
- ☐ Never

13. Currently, how often would you say you come into contact with sheep?
- ☐ Daily
- ☐ Once or twice a week
- ☐ Once a fortnight
- ☐ Once a month
- ☐ Once a year
- ☐ A few times a year
- ☐ Never

14. How much time have you spent working with sheep in your lifetime?
- ☐ Never
- ☐ A few occasions
- ☐ A few days
- ☐ A few weeks
- ☐ A month or more
- ☐ A year or more

15. Do you currently have any of the following as pets (tick all that applies):

☐ Dog(s) ☐ Cat(s) ☐ Bird(s) ☐ Fish ☐ Horse(s)

☐ Livestock ☐ Reptile(s) ☐ Rodent(s) ☐ Other

16. How often do you eat the following:

	Poultry	Lamb	Beef	Fish	Eggs	Dairy
Never	☐	☐	☐	☐	☐	☐

Occasionally (Less than once a week)	☐	☐	☐	☐	☐	☐
Often (1–4 times a week)	☐	☐	☐	☐	☐	☐
Always (> 5 times a week)	☐	☐	☐	☐	☐	☐

17. What factors do you consider when eating the following:

	Poultry	Lamb	Beef	Fish	Eggs	Dairy
Vegetarian	☐	☐	☐	☐	☐	☐
Religion	☐	☐	☐	☐	☐	☐
Dietary intolerance	☐	☐	☐	☐	☐	☐
Dislike product	☐	☐	☐	☐	☐	☐
Animal welfare concerns	☐	☐	☐	☐	☐	☐
Food safety	☐	☐	☐	☐	☐	☐
Cost	☐	☐	☐	☐	☐	☐
Other………………………………	☐	☐	☐	☐	☐	☐

18. Do you believe you purchasing animal welfare friendly products can positively influence animal welfare?

No　　　　Don't know　　　　Yes

Comments

19. If you consume meat, dairy or eggs, are you personally responsible for the purchasing these products?

☐ Yes ☐ No ☐ Not applicable

Comments

20. Do you believe animal welfare should be improved in Australia?

No　　　　Not sure　　　　Yes

Comments

21. What factors influence your assessment of animal welfare?

☐ Media
☐ Education
☐ Personal experience
☐ Family/social/peers
☐ Religion
☐ Not sure
☐ Other

Comments

22. As a consumer, would you be willing to pay more for products coming from facilities that are enhancing welfare beyond current industry standards?

☐ Yes ☐ No

Comments

23. How do you rate the welfare of **sheep** in Australia?

Poor　　　　Excellent

____

24. How do you rate the welfare of **sheep during road transport** in Australia?
Poor Excellent

____

25. How do you rate the welfare of sheep during **live export by sea**?
Poor Excellent

____

26. How do you rate the welfare of Australian exported sheep at their foreign destination?
Poor Excellent

____

Section 2 of Questionnaire**:** Your Unique Code is: __________________

1. I can tell how a group of sheep are feeling by the way they behave

   (*please put a dash on the line at the appropriate place)*

   Strongly disagree Strongly agree

   ____

Comments

2. I believe animal welfare is important
   Strongly disagree Strongly agree

   ____

Comments

3. I am influenced by the media in my perceptions of animal welfare
   Strongly disagree Strongly agree

   ____

Comments

4. Sheep show visible behavioural responses
   Strongly disagree Strongly agree

   ____

Comments

5. It doesn't matter what a person's background is, they will be able to tell how sheep are feeling
   Strongly disagree Strongly agree

   ____

Comments

6. People who don't eat meat are better able to interpret how sheep are behaving
   Strongly disagree Strongly agree

   ____

Comments

7. People who have pets are better able to interpret how sheep are behaving
Strongly disagree Strongly agree

____

Comments

8. People who have no preconceived ideas of sheep behaviour are better able to interpret how sheep are behaving
Strongly disagree Strongly agree

____

Comments

Supplementary Materials S2: Vehicle driver questionnaire.

Driver Questionnaire

Drivers involved in multiple journeys are only asked to complete Part B once

**Driver details**	
Gender	
Age group	Less than 30 31 – 50 51 – 70
Journey number that day	
Experience in driving (year)	
Total number of hours worked that day (at completion of trip)	

1. What do you believe should be the most important priorities for improvement of the livestock transport industry?
2. In terms of animal welfare during road transport, do you believe:
a. Improvements are needed
b. No improvements are needed
3. If answered (a), then what do you think are the most important animal welfare issues that need to be addressed during road transport?
4. Is the monitoring of sheep with respect to animal welfare during road transport adequate?
☐ Yes ☐ No ☐ Don't know
5. If no, how do you believe the monitoring could be improved?
6. In your experience do you believe you are able to tell how a sheep is feeling (e.g. stressed, content, fatigued) by the way it behaves?
☐ Yes ☐ No ☐ Don't know
7. If yes, do you think you are able to tell if a sheep's welfare is compromised by the way it behaves?
☐ All the time ☐ Some of the time ☐ None of the time ☐ Don't know
8. What specific behaviours would indicate to you that a sheep's welfare is compromised?
9. It doesn't matter what a person's background is, they will be able to tell how an animal is feeling by the way it behaves
☐ Yes ☐ No ☐ Don't know
Comments

Supplementary Materials S3: Transport event questionnaire.

Details of transport event

Journey Details	
Journey code	Date (0101):<br>Day journey number for that driver (01):<br>Driver initials (CS):<br>**CODE:**
Date	
Time of departure	
Place of departure	
Odometer at departure	
Odometer at arrival	
Place of arrival	
Time of arrival	
Number and duration of stops	
Comments/reasons for stops	
Weather conditions	
**Vehicle details**	
Type of vehicle	
Year of manufacture	
Position of cameras	**Diagram**
Time camera turned on	
Successful recording (circle)	Yes No
Use of dog (circle)	Loading Unloading
Placement of dog in front crate?	
Use of prodder	Loading Unloading
Cleanliness of vehicle (comment)	
**Experimental animal details**	
Breed	
Sex	
Age	

Average Weight	
Total stock on vehicle	
Number of stock in pen	
Approx size of pen	
Place of origin	Feedlot Station Pasture managed Other
Curfew	Feed curfew (circle) yes no<br>Time off feed before loading:<br>Water curfew (circle) yes no<br>Time off water before loading:
Cohort details	Are all experimental sheep from same location yes no<br>If no, detail:<br>Cohort number (in order of loading) and approx number from each :
